# Elucidating the modified performance of high nuclearity of Cu nanostructures-PTFE thin film

**DOI:** 10.1038/s41598-023-45478-5

**Published:** 2023-10-23

**Authors:** Nurul Akmal Che Lah, Puhanes Murthy

**Affiliations:** 1 Center for Advanced Intelligent Materials, Universiti Malaysia Pahang Al-Sultan Abdullah, 26300 Gambang, Pahang Malaysia; 2 Faculty of Manufacturing and Mechatronics Engineering Technology, Universiti Malaysia Pahang Al-Sultan Abdullah, 26600 Pekan, Pahang Malaysia

**Keywords:** Materials science, Nanoscience and technology

## Abstract

The aim of this study is to attain an extensive insight on the performance mechanism that is associated with the formation of Cu nanostructures- polytetrafluoroethylene (PTFE) thin film. The work presented Cu nanostructures synthesised via microwave-assisted method at different Cu precursor concentrations to observe the influence of different average particle diameter distribution, ${d}_{m}$ of Cu nanostructures on the fabricated Cu nano thin film. The thin films of Cu nanostructures with a layer of PTFE were fabricated using the Meyer rod coating method. Evaluating the effect of Cu nanostructures at different ${d}_{m}$ with overcoated PTFE layer showed that the resistance of fabricated thin film coated with PTFE is not significantly different from that of the uncoated thin film. The results implicate the influence of the PTFE layer towards the output performance, which can maintain a stable and constant resistance over time without affecting the original properties of pure Cu nanostructures, although some of the Cu nanostructures seep into the layer of PTFE. The novelty of this study lies in the effect of the intrinsic interaction between the layer of Cu nanostructure and PTFE, which modulate the performance, especially in photovoltaic cell application.

## Introduction

Adopting conductive high nuclearity pure copper (Cu) nanostructures ink as an alternative to carbon-based materials in printed flexible hybrid electronics to fulfil the worldwide market demand is rapidly accelerating^1–5^. Utilising high nuclearity Cu nanostructures as the nano inks for circuit-forming materials and bonding materials apart from other precious metals (*e.g*., silver and gold) has embellished not only because it is less expensive but also due to the high bulk thermal conductivity of ~ 398 W·m^−1^·K^−1^^6–8^ and other various factors (*i.e*., corrosion resistance, aesthetic appearance, and antimicrobial properties). The optimisation of these smart thin films in the integrated circuit production technologies to fabricate on-demand devices fundamentally relies on the enhanced electrical quantum conduction properties of those high nuclearity Cu nanofilaments and nanocontacts bonded to flexible substrates. Those portfolios of robust solution-processed Cu nanostructures (*e.g.,* Cu_1_, Cu_2_, Cu_3_, Cu_3_O, Cu_3_O_2_, Cu_4_, Cu_4_O, Cu_4_O_2_ and Cu_4_O_3_) should be suited and conformable to different surfaces, substrates, and geometries with other arbitrary form factors^9–13^.

Typical solution-synthesis nanostructure technique in achieving precise size control is one of the needs to fulfil the true solution processing that is compatible with either dip coating or roll-to-roll fabrication, which is usually confined to a soft thin film substrate. Despite massive efforts by researchers, most of the techniques yet endured deficient in antioxidation shielding, complex synthesis process, or uneconomical oxidised Cu nanosuspension, restricting their wide industrial applications. The ascending usage of the microwave energy source for the synthesis of pure Cu nanostructures has excellent superiority in accomplishing short times volumetric heating (in minutes), high phase purity and enhanced reaction kinetics, thus greatly minimise oxidation without the request for extra reagents^14–17^. The energy of microwaves is high mainly due to the longer wavelength of light that causes a more profound penetration ability. Hence, the applicability to conjugated or attached any reagents dependent under the uniform heating-dry circumstance is superior, plus producing narrow (consistent) particle size distribution and shape. The particle size distribution, amount of content and efficiency of produced high nuclearity Cu nanosuspension are hugely influenced by the simultaneously optimised parameters such as the microwave power, volume of solvent, time of reaction as well as the concentration of stabiliser^18–20^. Therefore, in view of these advantages, the microwave-assisted technique is the focused of this work in the aim to simultaneously reduce the costs, increase activity and enhance stability in which to realise the industrialised production of Cu nanostructures in the GHz range.

The use of Cu nanostructures is also reported as one of the promising functional nanoscale electronic materials owing to their unique optoelectronic properties^21–24^. In particular, the scalably liquid production of Cu nano solution makes it easy to create self-assembled flexible thin films and hybrid functionalities, thus suitable for current state-of-the-art device applications. Cooperating Cu nanostructures onto flexible and light thin film requires several crucial steps to endow the necessary structure performance such as drop cast, printing and painting that subsequently precede with drying or curing processes at certain elevated temperatures. A promising scalable and low-cost approach is via Mayer rod nano printing, which utilises the liquid characteristics in the manual patterning process of the delicate substrate at ambient temperature. Nonetheless, despite these significant advantages, an additional process is required not only to enhance the area utilisation rate but also to hinder the catastrophic breakdown of the thin film because of the compressive strains built between the nano paste and substrate during coating deposition with upraised temperatures. Uniform topography, a solid, tight and reliable bonding between Cu nanopaste and substrate are obligatory toward long-term cycling stability, high-rate and endurable electrical properties in flexible thin film devices. The states at these interfaces are indispensable for the behaviour of any device. Also, the constriction in preventing oxidation is to employ transparent passivated layers to improve connectivity between the Cu nanopaste and to compromise excellent conductivity regarding high transparency to visible light using either conducting oxide, conducting polymer, 2D organic nanotube and 3D oxide nanomaterials. In this case, accurate strategies using the polymer-based layers should not interfere with the electron pathways of the electrode nanostructures as the current collectors to avoid degradation of the energy density from the inactive electrochemical reaction. The operation stability includes temperature and hydrophobicity capabilities using a polytetrafluoroethylene (PTFE) coating as a passivation layer able to protect the nanoelectrode of the thin film while retaining good flexibility. PTFE is economically viable and environmentally safe (*i.e*., primarily used in medical applications) with a higher hydrophobic layer surface^25–27^.

The insight of overcoming the common challenges in Cu-based nano thin film is to compromise the high transparency and maintain low sheet resistance. Since the conductivity of Cu is known to be lower than Ag, it has been used primarily on flexible low-cost organic devices such as wearable electret generators. Although many reports have proven the capability of low sheet resistance (13.4 Ω/sq) and high transmittance of the hybrid Cu nanosuspension^28–30^, but limited references have reported the pure single-element Cu nanosuspension passivated by the PTFE layer to improve the stability of the thin film, which yet to exhibit some assurance in flexibility but fall short to bestow exceptional performance. The effects of the PTFE layer on the output performances of thin film structures have remained elusive. In this approach, the thin layer of PTFE is deposited via painting using the Mayer rod method^31–33^, which is able to conserve the indigenous polymer functionality on the surface substrate and to keep a low substrate temperature imperative to treat its mild deposition systemic conditions, permitting the deposition on flexible organic substrates.

Herein, the present work envisages bolstering the interfacial effect throughout the film and the functional Cu-PTFE nanolayer thin film. The work employs a microwave-assisted method to specifically synthesise the immobilised high nuclearity Cu nanostructures and the low-cost fabrication approach to fabricate PTFE functionalised-Cu nanostructures on PET thin film substrate. The optimised conductivity and optical properties are compared. The importance of the present work is not only in the successful fabrication of the thin film but also in exploring the variable influence to fine-tune the attributed properties, which could potentially be integrated into ubiquitous flexible electronics.

## Materials and method

### Materials

All materials were commercially available and used without any additional purification. Copper (II) sulfate (CuSO_4_, anhydrous, powder, ≥ 99.99% trace metals basis) as a source of the precursor was purchased from Merck (Malaysia). Polyethylene glycol (PEG, MW = 8000) acts as a reducing agent and polyvinyl propylene (PVP, MW = 5000) as a stabiliser were obtained from Alfa Aeser (Malaysia). Ethylene glycol (HOCH_2_CH_2_OH, reagent plus, ≥ 99%), the solvent is purchased from Merck (Malaysia). Polytetrafluoroethylene ((C_2_F_4_)n, powder, mean particle size 6–9 microns, 100 g) is purchased from Merck (Malaysia).

### Synthesis of Cu nanostructures

The synthesis procedure for preparing high nuclearity Cu nanostructures via microwave-assisted method is as follows. Initially, 2 g of CuSO_4_·5H_2_O powder was first dissolved in 20 ml of distilled water with stirring at room temperature under an air atmosphere. A similar step was repeated for 3 g of PEG and 3 g of PVP. The mixture was further processed using the conventional microwave oven with a 2.45 GHz frequency. The reaction starts with the dilution of CuSO_4_·5H_2_O into 90 ml of EG solution and is left for reaction in the microwave for 2 min. Subsequently, the diluted PEG solution was added to the mixture and the reaction continued for another 2 min. The colour changes with sediments were observed from bright blue to light blue. Next, the diluted PVP was mixed into the solution for 2 min and the colour was maintained as light blue. The solution colour changed after the reaction time increased for another 2 min, with the sediment colour changing from light blue to turquoise blue. The total reaction time for the microwave-assisted approach is 8 min. The experiment was repeated for a similar reaction time under the same environment but with a different amount of Cu precursor: Sample 2 (S2) contained 3 g and Sample 3 (S3) was filled with 4 g. Sample 4 (S4) condition is similar to S3 but with the combined reactions of hydrothermal and microwave-assisted methods. A summary of the preparation amount of chemical reactants is shown in Table 1. The solution was kept at room temperature to cool down the sample upon the reaction. The samples were washed with distilled water using a centrifuge to remove any impurities present during the reaction. The clean samples in distilled water slurry (approximately 20 wt% Cu) were then kept in the glass vial. Next, the samples were ready for characterisation.

**Table 1:** Parameters used in preparing high nuclearity Cu nanostructures via microwave-assisted technique and wt% of Cu nano solution made for thin film fabrication.

Sample	CuSO_4_·5H_2_O (Precursor) (g)	Concentration of the solution (mol/L)	pH	Cu Suspension (wt%)
S1	2	0.05	6	40
S2	3	0.08	6	40
S3	4	0.11	6	45
S4	4 (20 min on hot plate + 8 min in microwave	0.11	6	60

The weights of PEG and PVP remain constant at 3g each. The total amount of solvent used in all sample reactions is also fixed at 90 and 60 ml for EG and distilled water, respectively.

### pH measurement

The as-synthesised samples were diluted for 30 µM in 10 ml distilled water for all spectroscopic measurements. The pH value of each solution is observed using the pH strip (Natural Care, Park City, UT). The pHs after the dilution are shown in Table 1.

### Fabrication of high nuclearity Cu nanostructure-PTFE/PET thin film

As regards to the fabrication of flexible PET-based thin film, the thin film is first washed using alcohol and then placed onto a non-movable clean board. Specific concentrations of Cu nanostructure solutions are prepared based on the formulated ratio mixing with EG solution followed by ultrasonication for 15 min to homogenise. Subsequently, 2 ml of Cu nanostructure solution was uniformly drop-casted via rolling technique onto the surface of PET thin film using the clean Mayer wire wound rod (12.7 mm diameter, 406.4 mm length, 0.25 mil of wire) at ambient condition (cleaned with isopropanol and ethanol (1:1)). This followed by the drying up to 50 °C in a vacuum oven to dry the Cu nanostructures layer for 6 h. Pure Cu nanostructures/PET thin film is fabricated. Afterwards, the PTFE solution (0.005 g diluted in 50 ml distilled water, 0.1 mg/mL) was overcoated on the Cu nanostructure/PET substrate using the identical process of Mayer rod technique and dried in a 50 °C vacuum oven with a ramp rate of 1 °C/min to permit the solvent to wholly evaporate. Finally, the flexible Cu nanostructure-PTFE/PET thin film is ready. The thickness of the Cu nanosolution and PTFE layer controls the film thickness. The summary of the details of each Cu suspension used in the fabrication is shown in Table 1.

### Methods

#### Morphological measurement

The morphology of the Cu nanostructures and the coverage of the Cu nanostructure-PTFE/PET thin film surfaces were examined using a field-emission scanning electron microscope (FESEM) and elemental analysis was performed using energy dispersive X-ray (EDX, LSM880-FESEM, Carl Zeiss AG, Germany) mapping with an acceleration voltage of 5 kV. The typical magnification of FESEM-EDX is in the range of × 50,000 to 100,000. For FESEM-EDX, the solutions were dropped onto the aluminium foil substrate and let dry for 12 h prior to the observation.

Cross-sectional FESEM image was taken using cryogenic focused ion-beam (FIB, FEI Helios NanoLab 650 Dual Beam FIB and SEM, Europe) to check thin film thickness. The dual beam is equipped with a Ga^+^ ion FIB milling machine operated at room temperature, set off with an accelerating voltage of 30 kV and final thinning performed at 0.23 nA (3 kV). The cross-section sample is prepared and covered by a 1.5 µm platinum (Pt) layer that is deposited by the dual Ga^+^ ion beam on the top of the area of interest before the FIB procedure for protection. Later, the thinning process of the sample is performed using decelerated voltages (from 30 to 2 kV) of Ga^+^ ion to reduce amorphisation. The image acquisition speed is approximately 20 s per frame, with the count rate equal to 2000 cps. The conditions of the samples are analysed further using the EDX element mapping and lining approaches.

The size and shape of the Cu nanostructures were captured using transmission electron microscopic (TEM, LEO Libra-120 Zeiss, Germany). Samples for TEM were prepared by dropping the suspension of the Cu-based oxide nanostructure onto a lacey carbon-coated grid specimen holder and dried under ambient conditions for 12 h. The TEM micrographs were obtained at different magnifications and further examined using Gatan Micrograph software.

#### Crystallisation measurement

The crystal phase system of the Cu nanostructures were performed using X-ray diffraction (XRD, Rigaku MiniFlex II, Japan) equipped with a detector using Cu-Kα radiation (λ = 0.154 nm, 30 kV, 15 mA) and scanned 20–80° at a speed of 1° min^−1^ in a powder diffraction arrangement. Also, the as-equipped dimensional detector was used to scan the crystal phases of the samples. The dense powder of the sample was prepared by drying the dropped solutions of suspension on the thin glass substrate. The sample was then left to dry in a vacuum cabinet for 12 h. The crystallinity percentage of the sample is calculated from the area of XRD peaks using the relationship of^34–36^:

1 $${\text{Crystallinity}} = \frac{{\text{Area of crystalline peak}}}{{{\text{Area of all peaks}} \left( {{\text{crystalline}} + {\text{amorphous}}} \right)}} \times 100$$

#### Surface measurement

The surface property of Cu nanostructures was characterised using Fourier transform infrared spectroscopy (FTIR, Perkin Elmer—Spectrum 100, United States) equipped with an attenuated total reflection (ATR) system accessory with a resolution of 4 cm^−1^ in the spectral range between 4000 and 700 cm^−1^ (mid-infrared) and three individual scans for each measurement. 2 mL of each sample was deposited onto the glass substrate surface and dried at room temperature for a day in a vacuum cabinet to remove excess suspension base deionised water solution. The IR radiation propagated along the sample placed on the ATR transparent crystal to attain the corresponding spectra with an average of 30 data scan acquisitions. Each sample was analysed three times, and all data were obtained in triplicate.

#### Macroscale contact angle measurement

The contact angle ($\theta ,$ CA) on the thin film is measured by employing the sessile drop method (Attension- Biolin Scientific, Sweden) at room temperature with relative surrounding humidity in the r30 to 60 RH% range. The thin film was placed on a flat stage and the central area of the thin film was positioned on the glass of the flat stage. The droplet volume of the deionised water (polar component) was precisely controlled (3 µl) using a micro-syringe needle. The needle was slowly lowered until the water droplet came into contact with the thin film, which then reached the static state and was gently raised. The CA measurement was set off in video mode using OneAttention software with standard frame captures of 12 frames per second. The relation between the interfacial free energies of the film-vapour ${\gamma }_{F}$, contact radius *r* and the $\theta$ effectively change the capillary energy surface ${F}_{c}$ based on^37^:

2 $$F_{c} \sim r^{2} \gamma_{F} (1 + \cos \theta )$$

#### Conductivity measurement

The cross-thin film conductivity was measured through the AC electrochemical impedance method. The prepared rectangular thin film with a size of 2 × 1 cm and PET substrate thickness of 0.5 mm is placed on the sample stage with the tungsten carbide of 4 point-probe head in contact with the thin film surface at a constant load for each needle of 10 g. The probe is linked to the source manager unit (SMU, Jandel, England) testing station to measure the resistance, which was performed in the frequency range of 10–50 Hz. Note that the resistance measured includes the electric resistance of the fabricated thin film and the contact resistance between the point probe and the thin film. To obtain the contact resistance for the stack thin film, bare PET, Cu nanostructures/PET and Cu nanostructures-PTFE/PET thin films were measured. A relationship between resistance and the types of layers of the stacks was established with the electrical resistance of the fabricated thin film based on the slope of total resistance vs. the number of stacked layers. Additionally, to retain a persistent test environment, the measurements were operating at room temperature and a fixed relative humidity in the range of 50–60 RH%. The cross-thin film conductivity (lateral conductivity $\sigma ,$ *S* cm^-1^) of one piece of the thin film was calculated according to the following equation^38^:

3 $$\sigma = \frac{1}{\rho }$$

with $\rho$ is referring to the resistivity in $\Omega$ cm obtained from:

4 $$\rho = R_{sh} t$$

where ${R}_{sh}$ is sheet resistance of thin film in $\Omega$ sq^−1^ with $t,$ the thickness. The ${R}_{sh}$ in $\Omega$ sq^−1^ is given by:

5 $$R_{sh} = \frac{\pi }{ln2}\left( \frac{V}{I} \right) \approx 4.53 \left( \frac{V}{I} \right)CF$$

with *CF* is known for the lateral correction factor for the probe. Meanwhile, Hall effect measurement is performed using HMS ECOPIA 3000 under magnetic field of 0.57 T with the current of probe of 10 mA to find the carrier concentration, mobility, resistivity and average Hall effect of the samples.

## Results and discussion

### Morphology properties of as-synthesised high nuclearity Cu nanostructures

**Figure 1:**
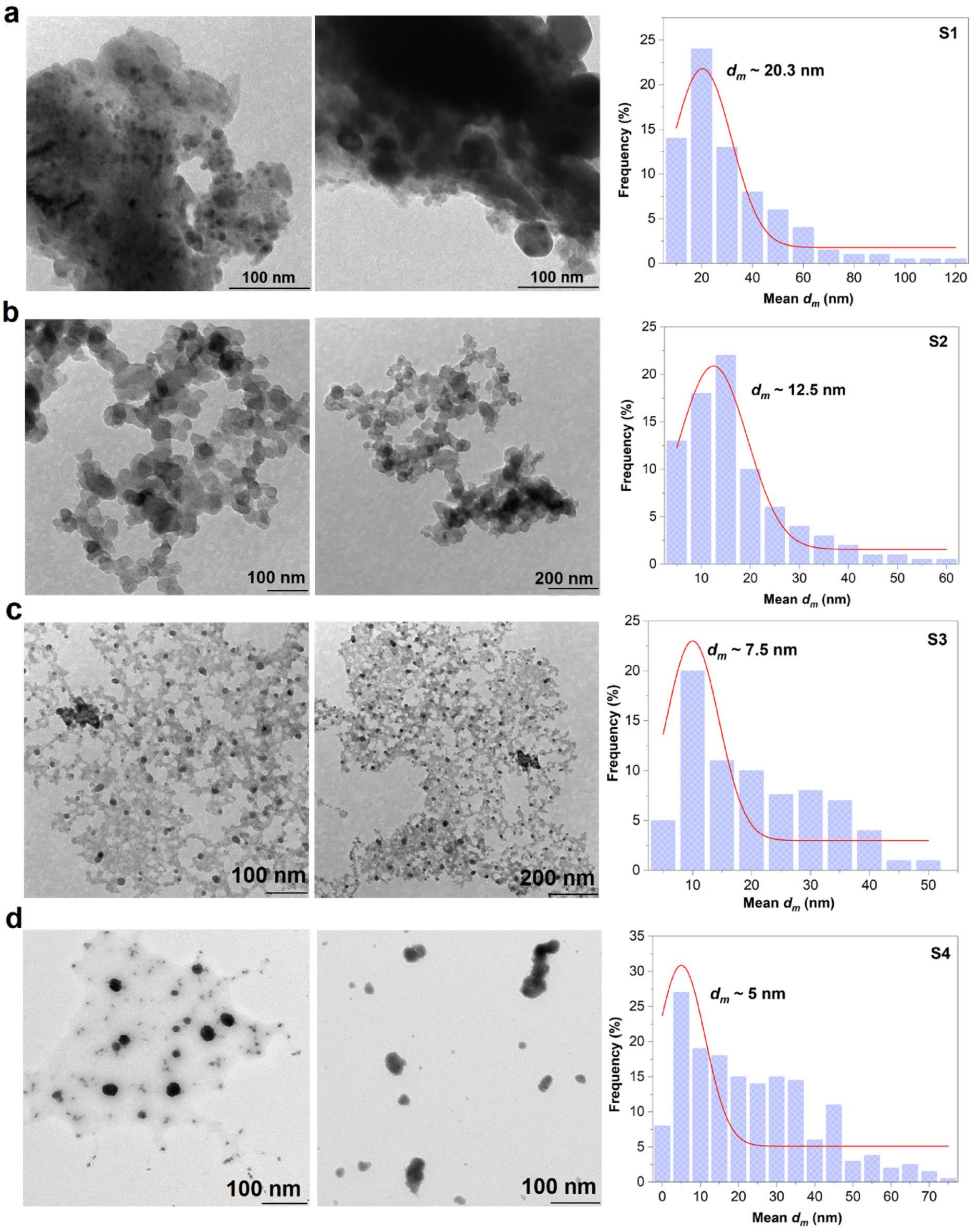
TEM morphology of the as-sythesised Cu nanostructures of (**a**) S1, (**b**) S2, (**c**) S3 and (**d**) S4 with their corresponding mean particle size distribution, ${d}_{m}$.

In order to confirm the morphology and mean particle size diameter, ${d}_{m}$ of the nanostructure, all the synthesised Cu nanostructures were observed and analysed using TEM. The corresponding ${d}_{m}$ denoted the nanostructures as sub-nanostructures with the ${d}_{m}$ histogram of 20.3 $\pm$ 1.4, 12.5 $\pm$ 0.6, 7.5 $\pm$ 1.4, 5 $\pm$ 2.2 nm for S1, S2, S3 and S4 as shown in Fig. 1a–d, respectively. The morphological and ${d}_{m}$ differences in nanostructure are presumed to be mainly caused by the different concentrations of metal Cu precursor used in the reaction, as evidenced in Fig. 1a–c. The growth rate primarily depends on the amount of Cu ion presence which further affects the adsorption of capping agents on the nanocrystal planes, leading to different morphology of nanostructures as observed in all samples. Since the PVP concentration is fixed, the morphology and shape changes are not significantly different. Most of the particles in all samples (S1, S2, S3 and S4) have nano octahedra crystals, which are then easy to generate larger particles once the Ostwald ripening process takes place. The morphology of the suspended Cu nanostructure solutions is found to be much more nearly identical to each other with anisotropic in shape. The effect of H_2_O oxidants in the reaction indicates the strong oxidising power of H_2_O that yields the endothermic (positively charged surface) formation energies that caused by the elevated temperatures for Cu_3_ and Cu_4_ clusters yielding the tetrahedron particle shape. The micrograph of S1 revealed the severe accumulation of particles, creating several nanocrystalline layers of Cu on top of the others as indicated in Fig. 1a. In S1, the aggregated size of Cu could not be precisely measured, in which the much smaller particles are observed to be trapped in larger pieces of Cu. This is confirmed by the broad particle size distribution when compared with ${d}_{m}$. On another note, as demonstrated in the micrograph of S2, the nanostructures grow perpendicular to each other, indicating the vast majority of nanonetwork branches and reinforcing contact between the ordered nano (Fig. 1b). As revealed in Fig. 1c, the S3 sample demonstrated moderate clustering of particles with polydispersed assembly at the highest Cu precursor concentration synthesised under microwave radiation. The trend of assembly suggests that S1, S2 and S3 samples show a propensity to agglomerate at low Cu precursor concentration. On the other hand, the smallest ${d}_{m}$ of highly dispersed Cu nanostructures is only observed in S4 after the reaction method takes place using the combined reaction method of the hot plate and microwave, as shown in Fig. 1d. The S4 image proves better uniformly distributed particles with extensive monodispersity of the particles, suggesting smaller nanostructures produced at the highest concentration of administered Cu precursor under a mixed reaction of hydrothermal and microwave irradiation methods.

Overall, the best explanation for this phenomenon is that the changes in ${d}_{m}$ caused by the changes in concentration of CuSO_4_·5H_2_O, the precursor powder. This is attributed to the increase of activation energy medium leading to the atomic grain transformation and the yield of much more coarsening nanostructures, which is controlled by the existence amount of the precursor apart from the heating reaction. It can be observed that with increasing the precursor concentration, there is a strong tendency for new nucleation of the atom, with some of the atoms being individually separated from each other. In contrast to the low precursor concentration system (S1 and S2), the amount of newly generated nuclei is lesser and it can be observed that the inclination for the existing particles to coalesce and connect to one another conjoin into big aggregate nanostructures is significant.

### Crystallisation and surface measurements

**Figure 2:**
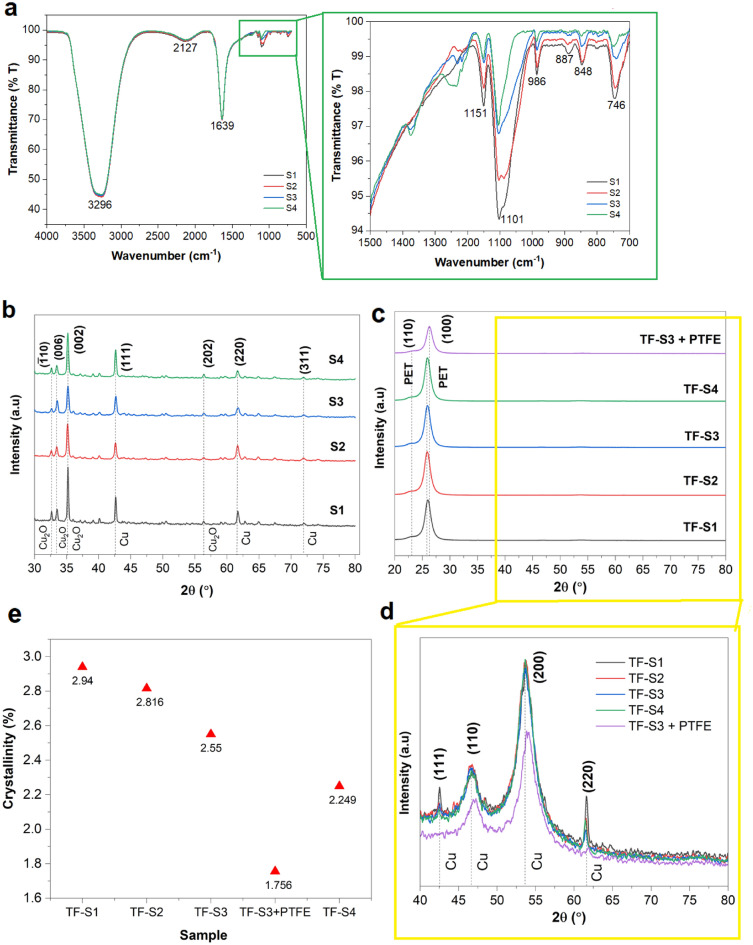
Surface analysis of (**a**) ATR-FTIR spectra of as-synthesised Cu nanostructure solutions with the inset showing the areas of 700–1500 cm^−1^. Characteristic vibrational features are mentioned and spectral regions are highlighted. (**b**) XRD spectra of Cu nanostructure solution and (**c**) XRD spectra of the fabricated thin film of Cu nanostructures along with Cu nanostructure/PTFE with the inset in (**d**) the magnified 2$\theta$ in the range of 40–80° showing the peak of Cu element. All the patterns and lattice parameters remained nearly similar across various Cu precursor concentrations. (**e**) Showing the % of crystallinity for each thin film sample based on the XRD data of (**c**) and (**d**).

The ATR FTIR spectra for the series of the as-synthesised Cu nanostructure solutions to demonstrate the probable interactions of Cu nano with the related functional groups are evinced in Fig. 2a. Overall, the spectra are very similar and resemble almost identical chemical compositions based on the present peaks. The most distinct and broad peak observed at ~ 3296 cm^−1^ corresponds to the free –OH stretching contributed by PVP, EG and H_2_O solutions that overlapped with each other. It is known that the intermolecular bond of hydrogens in EG is much frailer compared to the hydrogen bonding in H_2_O that generates the stable tetrahedral molecule. Hence, the tendency of the H_2_O molecule to form a hydrogen bond with EG is much superior and distorts the –OH–OH bond of EG. A similar condition was observed for PVP in which, theoretically, the –OH band of PVP is much lower in intensity, with the peak being weak and broad. A minor broad peak with low intensity is observed at ~ 2127 cm^−1^ attributed to the C=O carbonyl stretching mode band representing a change in the dipole of the polarisation created Cu element. The second intense band around ~ 1639 cm^−1^ corresponds to the C=O absorption peak from the amide I group of PVP. The C=O uptake the moisture adsorption in PVP that could also cause the brittleness of the PVP layer. A number of literatures have demonstrated strong hydrogen bonds by the presence of a C=O stretching band in PVP below 1664 cm^−1^. This suggested that the PVP is embodied and accumulated into a micellar structure with Cu nano as the outer layer. In the inset, the band positions in the ~ 700 to ~ 1500 cm^−1^ area are similar in all samples but with a far reduction of the transmittance at this region in the case of the spectrum of nanostructures. The PVP-Cu characteristic peak assigned to C–N stretching vibrations of aliphatic amine in the regions of ~ 1151 cm^−1^ suggests coordination between them. The peak at ~ 1101 cm^−1^ shows the existence of –COO carboxylic aromatic acid interaction of EG with Cu nano. The band observed at ~ 986 cm^−1^ is assigned to C–C in-plane bending, which deviates from the bonding of PVP and Cu nano. In the case of in-plane bending –CH_2_ double band at ~ 887 and ~ 848 cm^−1^ are assigned to rocking vibration due to the mild interactions between the EG and PVP with Cu. All these showed that the EG might tend to absorb onto the Cu nanostructures, whereas the PVP is likely to desorb and leave the surface. The PVP is the capping agent in the synthesis and helps in forming the soluble molecular of Cu nanostructures.

The distribution of Cu elements in the as-synthesised Cu nanostructures and the corresponding thin film-based Cu nanostructures confirmed by the XRD are shown in Fig. 2b–d. It is worth noting that the heating to evaporate the samples might have influenced the elements obtained from the test specifically for the Cu nanostructures sample solution. The reflections of Cu nanosolution shown in Fig. 2b are assigned to both the pure Cu and CuO crystal phases indicated by the diffraction planes. The reflections corresponding to Cu (111), Cu (220) and Cu (311) planes detected at 2$\theta^\circ$ of 42.6$^\circ$, 61.5$^\circ$ and 71.9$^\circ$, respectively, in all samples. Meanwhile, the peak at 2$\theta^\circ$ of 32.6$^\circ$, 33.5$^\circ$, 35.1$^\circ$ and 56.4$^\circ$ specified for Cu_2_O in which due to the fact that using a weak reducing agent that does not mitigate all the Cu_2_O species. The weak reflection of Cu phases compared to CuO is ruled out due to the handling and XRD preparation sample reasons, as the samples are dried under direct heat and further exposed to ambient air when transferring for test purposes. Also, the possible reason is due to the small ${d}_{m}$ with diameter of less than 10 nm as a huge population of particles exist in all samples and may have lower crystallinity which cannot be ruled out on this measurement.

The recorded XRD spectra on Cu nanostructured thin film samples to investigate any structural changes in the system are shown in Fig. 2c. Interestingly, no substantial change in the XRD pattern for all thin film samples indicates that the sharp and separated pattern was not concerned with any interface effect but merely due to the deposited nanomaterial itself. Therefore, the structural planes are the same for all thin film samples. As can be seen, XRD of the PET thin film substrate demonstrates almost exclusively one single peak referring to the (100) and (110) planes without any obvious peak of the Cu nanostructures phases on the PET surface. Therefore, compared with the well-aligned direction of PET (100) and (110), the weakened XRD peaks of the Cu nano deposited on the PET thin film are not clearly observed. Nonetheless, the observed Cu nano peaks as exhibited in the magnified plotting of Fig. 2d, indicate very low intensity in relative to PET substrate peaks. This also considering the differences in their level of crystallinity and thickness of the fabricated thin film samples. Interestingly, it shows no typical Cu_2_O peaks and the only Cu phases are observed which is also considering the prepared thin film sample, although it has not undergone heat treatment during the fabrication, the phase change of Cu still occurred. This might be due to the loss of oxygen atoms upon reaction with hydrogen from the surface dynamic of PET in the presence of water, which can lead to a change of Cu_2_O to pure Cu^39,40^. The presence of several different lattice parameters of pure Cu is the result of their self-arrangement on the connected void of the PET porous structure. In comparison to the peaks obtained in Cu nanostructures solution samples, the intensity of all Cu peak of the thin film samples is weak (very low intensity), indicating poor crystallinity of the deposited Cu on the samples and most of the Cu domains deposited on PET surfaces are aligned in Cu (200) direction. Further experiment is needed in the future to prove the point.

Here, in the analysis of behaviour in a thin film system, the changes in the height of the XRD intensity peak are associated with the magnitude of change in per cent change in defect density and peak shifting, $\%\mu$ directly caused by the variation in the amount of Cu presence^41^. The theory of $\%\mu$ is fundamentally relies on the peak shift towards a lower angle. In this case, the peak of PET influences the overall intensity of the thin film and the signifies peaks shifted to a lower angle (or higher crystallite size value), reflecting larger crystallite radii of PET^42^. In essence, the increases of $\%\mu$ are related to the decreases in the degree change of their crystallinity. The increase in $\%\mu$ resulted from the larger crystallite size and generally led to improved overall properties of the thin film. Nonetheless, the surface area of each thin film is inversely proportional to the % of crystallinity, with the theory that indicates very high crystallinity films possess the least surface area and vice versa^43–45^. Thus, the % crystallinity of the as-synthesised deposited Cu samples plotted in Fig. 2e demonstrates that the deposited layers of Cu have very low crystallinity (1.76–2.94%) with respect to the porous PET substrate, which is confirmed by the weak diffraction pattern of the corresponded element beforehand. Once the Cu nanostructure suspension is rolled on the surface of PET, it reacts with the PET surface at room temperature and tends to adjust into the porous structure and create new orientations with respect to the PET substrate. PET substrate shows two corresponding peaks, (110) and (100), with (100) is broad, contributing to the amorphous region. Amorphous regions give PET toughness, thus being able to flex and bend without easily rupturing. This proposes that smaller crystallite size in the low crystallinity films (too small to detect) provides desirable smoother surface^46^ and enhanced surface area for Cu nanostructures/PET interactions and intermolecular forces, thus appreciable thin film solar sensing responses. Also, a high surface energy substrate is a choice for limiting the probing liquid such as water.

### Assemble layer of thin film

**Figure 3:**
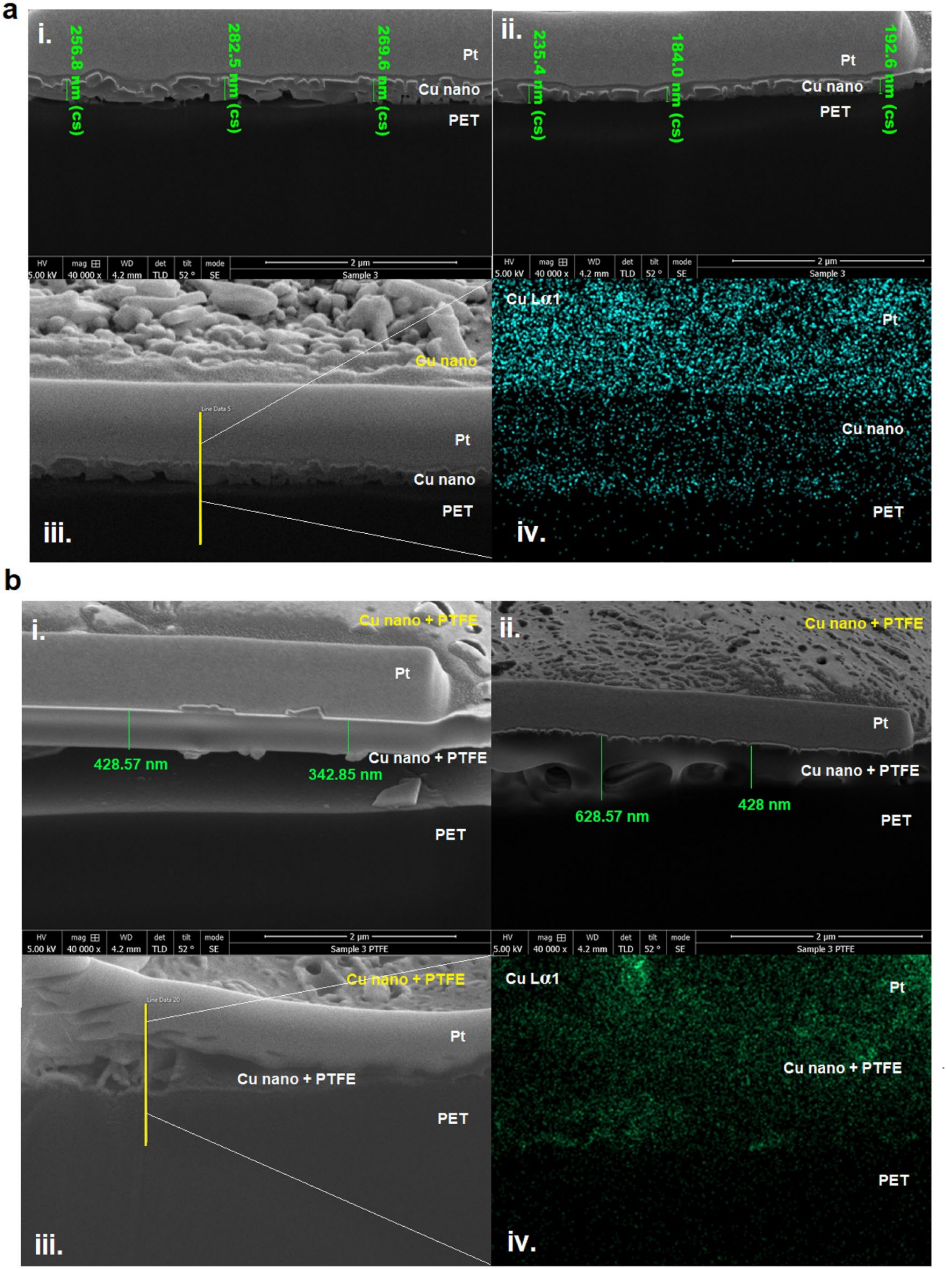
Cryogenic FIB-FESEM images of cross-sections for (**a**) bare Cu nanostructures/PET (TF-S3) (**i** and **ii**) and (**b**) Cu nanostructures-PTFE/PET (TF-S3 + PTFE) (**i** and **ii**) thin films with platinum (Pt) layer pre-coating on the top for protection. The scale bar is 2 µm, as indicated in the images. The corresponding FIB-EDS images and its mapping of the bare Cu nanostructures/PET (**a** (**iii** and **iv**)) and Cu nanostructures-PTFE/PET (TF-S3 + PTFE) (**b** (**iii** and **iv**)) in the thin films is included.

The cryogenic FESEM-FIB samples for both bare Cu nanostructures/PET (TF-S3) and Cu nanostructures-PTFE/PET (TF-S3 + PTFE) thin films are shown in Fig. 3. The cross-section images indicated the presence of organised Cu nanostructures thin layered (Fig. 3a (i and ii)) that is distinct from the assembled particles and layers of Cu nanostructures-PTFE thin film (Fig. 3b (i and ii)), which are found to be misoriented with respect to their respective layers. As indicated in the figures, the Cu nanostructures layer is made by rolling the solutions to fabricate two distinct layers of Cu nano and PTFE thin films. A similar method was used for the bare Cu nanostructure layer. Clearly, the layer of PTFE is mixed with the layer of Cu nanostructures and the thickness of adsorbed PTFE on the surface of the Cu nanostructures is varied. This creates inconsistent lamella thickness with random orient structures of the coated layers as indicated in the FIB-EDS images (Fig. 3a(iii) and b(iii)) taken from the yellow line and their corresponding mapping images (Fig. 3a(iv) and b(iv)). The tendency of the PTFE molecule to dominate and the notable density difference between polymer and metal leads to difficulty separating the PTFE layer from the metal layer; thus, the large thickness is estimated. The aggregation of PTFE impedes the consolidation of the individually layered composites by which the Cu nanostructure is absorbed onto the surface of the PTFE molecule, although the layer-by-layer assembly is used. Still, the main obstacle can be overcome by increasing the layer thickness of Cu nanostructures to balance the bulk amount of the PTFE molecule layer.

**Figure 4:**
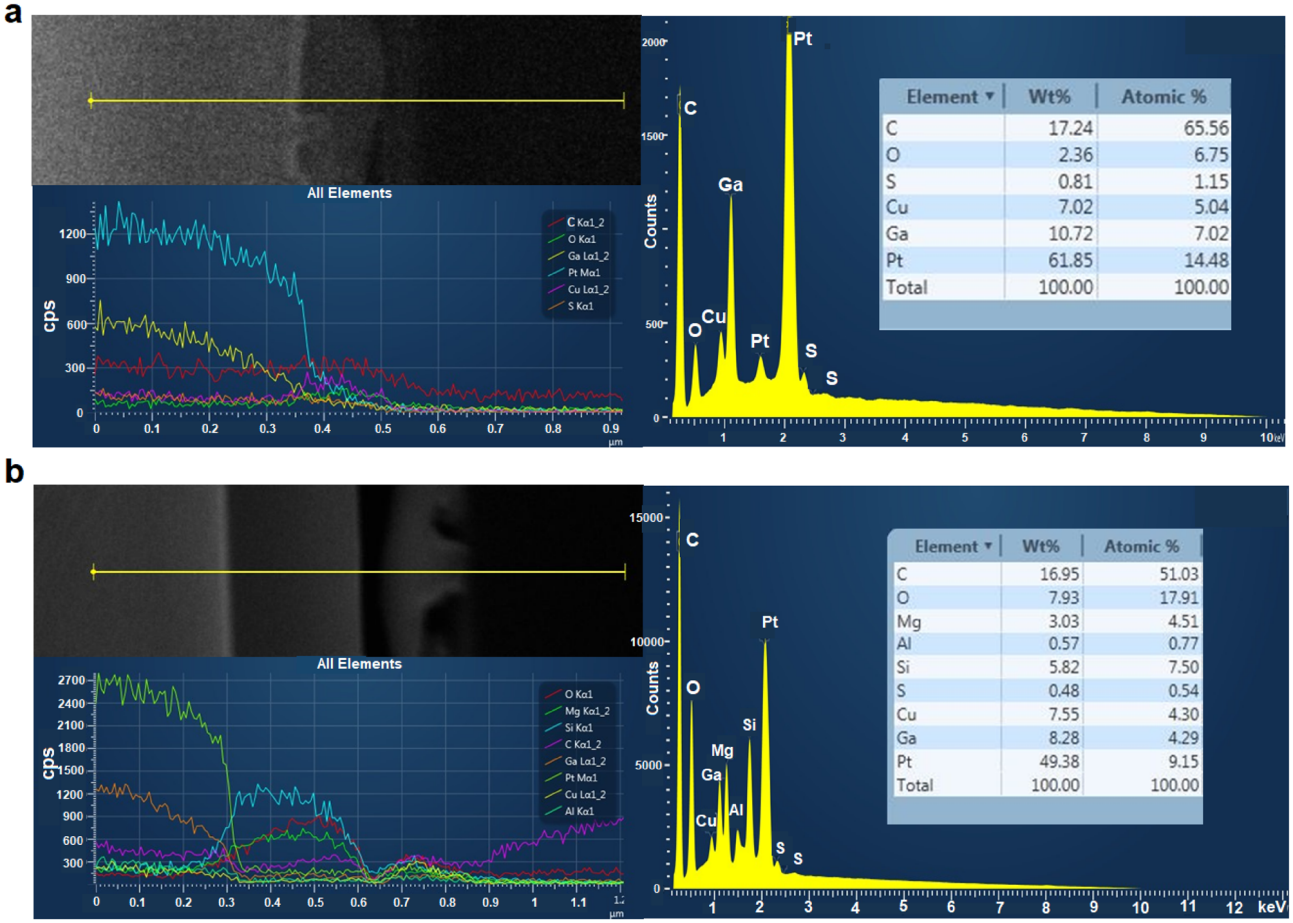
FESEM-EDX line profiles (left) based on the EDS line scans (along the yellow line – top left) with EDX spectra (right) of those respective area lines from micrograph image illustrating the element constituent concentrations presence in (**a**) bare Cu nanostructures/PET and (**b**) Cu nanostructures-PTFE/PET thin films.

The FESEM-EDX line (left) revealed the presence of the Cu nanostructure (Fig. 4a) and Cu nanostructures-PTFE (Fig. 4b) layers with other component elements that exist on the PET surface. For this measurement, it is clearly shown that the Cu line profile is reduced in intensity for bare Cu nanostructured thin film (270 ± 120 CPS; red line) compared to the Cu nanostructures-PTFE (360 ± 190 CPS; yellow line) thin film, suggesting perfect isolation of Cu layer from the PET surface for bare sample. Note that this intensity represents the thickness of the adsorbed layer and is calculated as a difference between the highest and lowest intensity peaks of those respective material signals. Since the Cu nanostructures-PTFE thin film in a mixed condition with the nano-size Cu is almost entirely adsorbed onto the PTFE layer, thus forms a thicker layer. The intensity of the oxygen, O layer (150 ± 40 CPS; green line) is among the lowest intensity apart from sulphur, S (120 ± 30 CPS; brown line) presence in bare Cu nanostructures/PET thin film which indicates the low oxide condition of the thin film. The weight per cent, wt% of Cu and O are shown as 7.02 and 2.36, presenting a stoichiometric ratio of Cu to O of 2.97:1, which is close to 3:1, indicating that the Cu nanostructures solution is oxocopper, Cu_3_O structures, the freshly prepared sample straight to fabrication without undergoing any heat treatment upon/during the synthesis/fabrication. Also noted that the H_2_O oxidation is the opposite of the H_2_ reaction and that yield the endothermic H_2_O reaction corresponds to an exothermic H_2_ reduction energy according to the equation ${Cu}_{x}O+ {H}_{2}\to {Cu}_{x}+{H}_{2}O$. Indeed, it shows that the oxocopper is less oxidised.

In contrast to Cu nanostructures-PTFE/PET thin film, the enriched intensity concentration of O element (780 ± 320 CPS; red line) is due to the effect of O bound with PTFE molecule that leads to reduced surface wettability angle (weakly water-wet with a CA of 87.5 $\pm$ 3.6° in Fig. 4a) compared to the reported literature for pure PTFE (CA of 108–115°). The trace of carbon, C in both samples originated from the carbon tape stub under the sample during the observation and thus, the signal from the stub easily penetrated the open or empty spaces in the samples, giving such a high value. Meanwhile, the Pt protects the sample from the immediate accumulation of electrostatic charging on the thin film surface as the Cu possesses electrical conductors and provides charge-free surface properties. By comparison, the quite intense trace amounts of silicon, magnesium, aluminium, carbon and silicon are presented and associated with the constituents of the PTFE in the Cu nanostructures-PTFE/PET thin film. These high-intensity level suggested that the PTFE bonds with the Cu nanostructures is strong and the larger size of PTFE particles is strong over the entire surface of Cu nano, which is consistent with their EDX chemical composition (Fig. 4b—right).

### Wetting angle property of thin films

**Figure 5:**
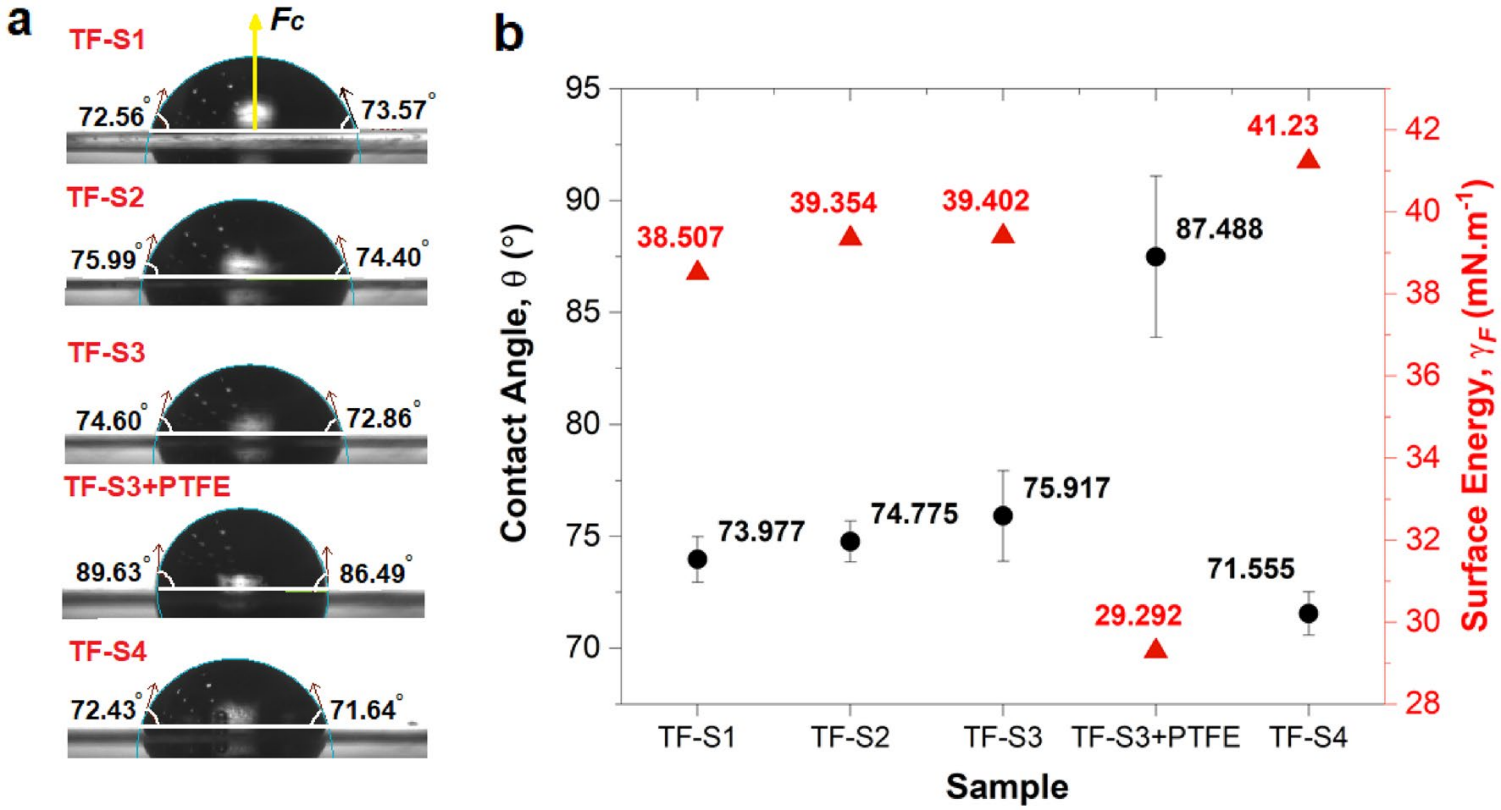
(**a**) Photographic images showing the comparison in the water droplet shape of static contact angle on the fabricated bare Cu nanostructures (TF-S1, TF-S2, TF-S3 and TF-S4) and Cu nanostructures-PTFE thin films (TF-S3 + PTFE). (**b**) The plots of apparent contact angle, $\theta$ of the water droplet and the surface energy, ${\gamma }_{F}$ of the four tested bare Cu nanostructures with one Cu nanostructures-PTFE/PET thin films.

The cross-sectional macroscale surface wettability images of the fabricated Cu nanostructures-PET and Cu nanostructures/PTFE-PET thin films are depicted in Fig. 5a. The static wettability photographic images for all samples show the low wetting range droplet shape with the water CA less than 90°, indicating the hydrophilic surface of thin film and indicated in the plotting of Fig. 5b. The static CA of Cu nanostructures thin film (TF-S3) is ca. 75.9 $\pm$ 2.0°, indicating the hydrophilic surface. The functional group of PEG and PVP, the highly hydrophilic polymers, promoted the changes in the microstructure and chemical component incorporated into the underneath Cu nano lattice, so the low water CA of the thin film is produced. As for comparison, the surface modification involved using PTFE on the Cu nanostructures layer (TF-S3 + PTFE), resulting in the static water CA of 87.5 $\pm$ 3.6°, the highest water CA. The slight increment in static water CA in the presence of hydrophobic PTFE is attributed to the contribution of the PTFE monomer structure layer that also depends on the PTFE thickness. Although the PTFE has low surface energy, the effect on the water CA implies only 11.6° differences due to the thin PTFE layer coating. The water CA follows the order of 87.5 $\pm$ 3.6° (TF-S3 + PTFE) > 75.9 $\pm$ 2.0° (TF-S3) > 74.8 $\pm$ 0.9° (TF-S2) > 73.9 $\pm$ 1.0° (TF-S1) > 71.6 $\pm$ 0.9° (TF-S4). The high CA in S3 in comparison to other bare Cu nanostructured thin films can be accounted for by the increase of the output yield with the decrease in ${d}_{m}$ of the as-synthesised Cu nanostructure that changes the surface feature. Although the CA indicates the low hydrophilicity or intermediate wet surface (70° < CA > 90°), it is worth noting that the Cu is naturally hydrophobic in bulk nature. This condition implies that the inherent slight hydrophilicity needs to be considered on a case-by-case basis since the present work used hydrophilic reagents to modify the surface, lessen the defects of as-synthesised Cu nanostructures gives the extensive ability to enrich the hydrophilicity of the nanostructures. On another note, defects provide active edge sites that enhance the free energy and hence guide towards refining the wetting capability, represented by the ranging crystallinity of Cu nanostructures that further modulate their wetting behaviour. Also, the hydrophilicity is sensitive to Fermi level changes and directly depends on the ${d}_{m}$ of the nanostructures, as noted in the previous section. The ${d}_{m}$ of S1 is the largest (20.3 $\pm$ 1.4 nm), which also indicates the number of air traps between the particles is decreased, then the air pocket will lose, resulting in the CA tending to be much more hydrophilic than the other smaller ${d}_{m}$. Therefore, the ${d}_{m}$ plays a crucial role in the surface wettability of thin film for solid nanostructures. Due to the nanoscale size structures, the tendency of water droplet penetration in between nanostructures apertures is high and that further substitutes the air pockets that exist in the structures, causing the different degrees of hydrophilic wettability type.

The reverse sequence is evidently regarded in the surface energy,${\gamma }_{F}$ of these samples. With the increase in the amount of Cu nanostructures yield, these direct ${\gamma }_{F}$ measurement shows low surface tension ($\theta <90^\circ )$ with slight increment from 29.29 (TF-S3 + PTFE), 38.5 (TF-S1), 39.3 (TF-S2) to 39.4 (TF-S3) and 41.2 mN.m^-1^ (TF-S4). These results demonstrated that surface wettability controls the CA and contact radius, *r,* which effectively changes the ${F}_{c}$ according to the Eq. (2). It shows that the S3 + PTFE thin film indicates the lowest ${\gamma }_{F}$ of 29.3 mN·m^−1^ with ${F}_{c}$ of 133.4 $\mu$N contributed by the disruptive intrinsic hydrophobic surface of PTFE. In a similar vein, the influence of the used solvent in the synthesis of Cu nanostructure solution dominates the overall disruption of samples’ intrinsic wetting behaviour. The calculated ${F}_{c}$ values for the thin films are tabulated in Table 2. Unlike the ${\gamma }_{F}$, there is no specific pattern observed for the ${F}_{c}$.

**Table 2:** Parameters to describe the capillary energy surface, ${F}_{c}.$

Thin film	${r}^{2}$(mm^2^)	cos $\theta$	${F}_{c}$ ($\mu N)$
TF-S1	2.83	0.149	127.9
TF-S2	2.87	0.812	200.3
TF-S3	2.54	0.868	186.9
TF-S3 + PTFE	2.36	0.888	133.4
TF-S4	2.58	− 0.763	25.1

The calculations are made for water droplets of all thin film samples from the measured parameters.

### Electrical and hall mobility property

**Figure 6:**
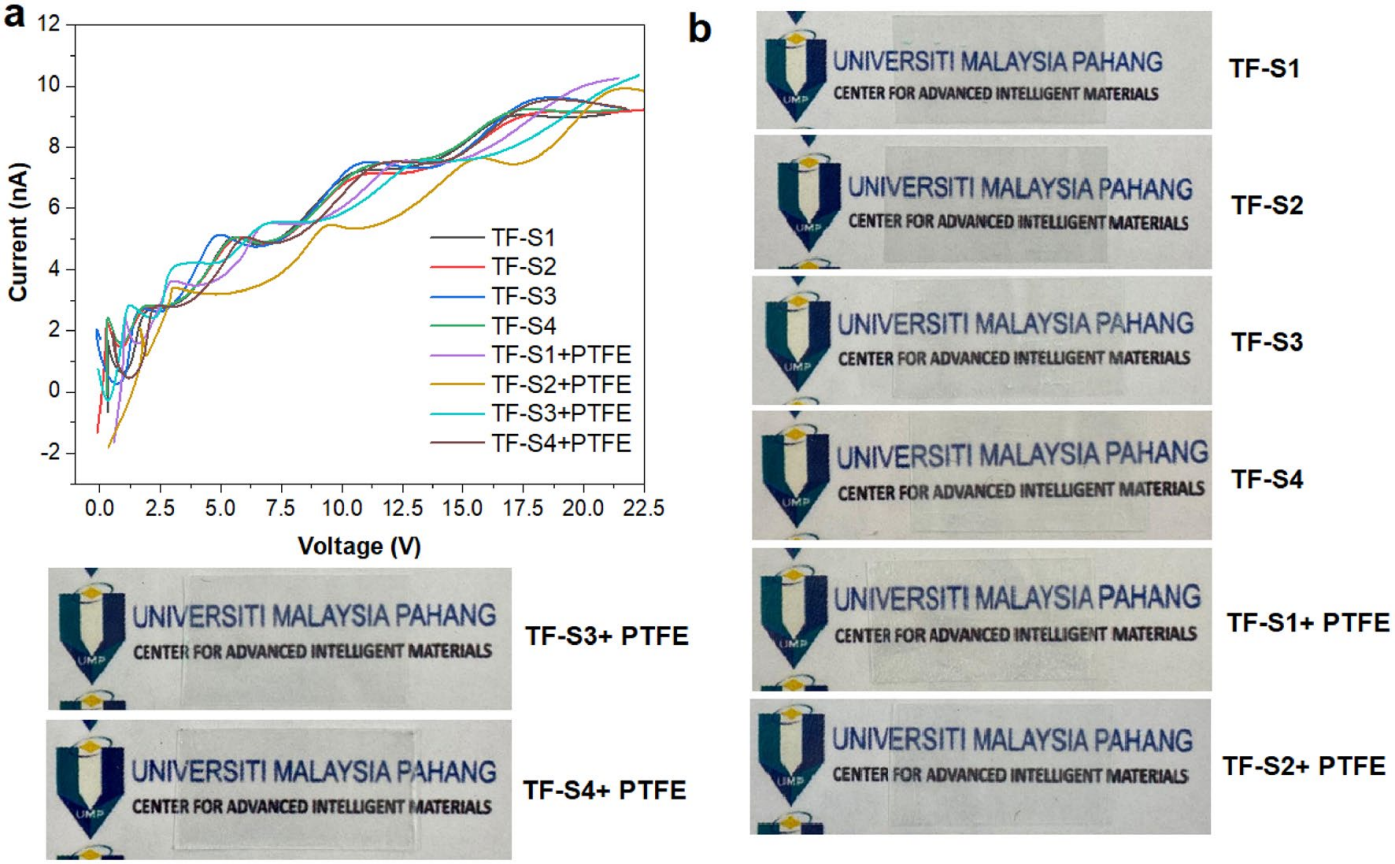
(**a**) The I–V measurement of Cu nanostructures-PET (TF-S1 to TF-S4) and Cu nanostructures/PTFE-PET (TF-S1 + PTFE to TF-S4 + PTFE) thin films. (**b**) Photographs of fabricated Cu nanostructures-PET (TF-S1 to TF-S4) and Cu nanostructures/PTFE-PET (TF-S1 + PTFE to TF-S4 + PTFE) thin films.

The performances of the fabricated Cu nanostructures-PET and Cu nanostructures/PTFE-PET thin films are compared based on the I–V curves measured by the four-point probe (lateral conductivity) shown in Fig. 6a. The straight-line implying accordance with ohm’s law, shows the metal characteristics of the thin film and provides the conductive pathway to improve the electrical conductivity. The corresponding lateral conductivity from I–V characteristics for each sample are summarised in Table 3. The process of reactions follows the conventional reaction of the Cu element. As the positive voltage is applied to the thin film samples, Cu ions are produced in relation to the oxidation–reduction reaction^47^:

6 $$Cu \leftrightarrow Cu^{ + } + e^{ + }$$

**Table 3:** The corresponding average sheet resistance, ${R}_{sh}$, resistivity, $\rho$ and conductivity, $\sigma$ measured from four-point probe.

Thin film	${R}_{sh}$ $({10}^{-9 }\Omega$ · sq^−1^)	$\rho$(${10}^{-7}\Omega$· cm)	$\sigma$ $({10}^{-8}S$ · cm^−1^)	${N}_{b}$(${10}^{14}$ cm^−3^)	${\mu }_{h}$(cm^2^ · Vs^−1^)	${\rho }_{h}$(${10}^{3}\Omega$ · cm)	Avg. Hall Coeff. (10^4^cm^3^ · C^−1^)
TF-S1	5.89	2.94	5.86	1.77	8.13	4.98	− 5.71
TF-S2	5.60	2.80	7.30	1.99	6.49	5.02	− 3.07
TF-S3	9.46	4.73	3.44	2.46	5.29	5.07	− 3.24
TF-S4	4.61	2.31	7.81	3.04	2.95	4.95	− 5.98
TF-S1 + PTFE	6.35	3.17	3.97	–	–	–	–
TF-S2 + PTFE	5.92	2.96	4.96	–	–	–	–
TF-S3 + PTFE	6.24	3.12	4.21	2.10	6.18	5.03	− 4.01
TF-S4 + PTFE	8.59	4.30	4.35	2.01	7.21	4.93	− 6.01

The carrier concentration, ${N}_{b},$ mobility, ${\mu }_{h}$, resistivity of Hall effect, ${\rho }_{h}$ and the average Hall effect coefficient obtained from Hall mobility measurement.

The reaction causes the migration of the Cu ions under the effect of electric field, creating more accumulation of Cu atoms. This condition yields higher protrusion because of the increase of Cu atoms and the metallic filaments (CF) of Cu atoms as the conductive path creating the conductance. The lateral conductivity present in Cu nanostructures-PET and Cu nanostructure/PTFE-PET is in the range of 3.44 to 7.81 $\times {10}^{-8}$ S·cm^−1^ and 3.97 to 4.96 $\times {10}^{-8}$ S·cm^−1^, in which Cu nanostructure/PTFE-PET possess much lower conductivity values. As noted beforehand, all samples are prepared at an air–water interface with the presence of non-ionic surfactant monolayers which proves to decrease the conductivity values. Note that the $\sigma$ values are good as compared to other reported bare Cu nanostructures without the addition of other metals^48^. For comparison purposes, it can be seen that the role of outer layer PTFE to increase the ${R}_{sh}$ although the increment is not significant. But the excessive amount of PTFE could lead to the decrement of the $\sigma$ due to their large molecule, which increases the contact resistance. This suggests that an appropriate amount of PTFE < 0.1 mg/mL to coat Cu nanostructures and enhance the conductivity of the system. That is, if PTFE suspension is < 0.1 mg/mL, the electronic properties purely depend on the concentration of Cu nanosuspension, not governed by the outer layer of PTFE. The simple Meyer rod method by layering the Cu nanostructures and PTFE on the PET for the same amount of Cu nanostructures but with different ${d}_{m}$ distribution indicates the different transparency level as shown in Fig. 6b. As the average ${R}_{sh}$ increase from 6.35 to 8.59 for Cu nanostructures/PTFE-PET thin films, the transparency is also decreased as compared to the bare Cu nanostructures-PET thin films. The low transparency is due to the PTFE layer that protects Cu nanostructures from scratching and corrosion resistance. This condition is ideal for solar cell application and desirable for photovoltaic systems.

Hall effect measurement across the interface is also carried out to further corroborate the properties of Hall coefficient based on carrier concentration, ${N}_{b}$, Hall mobility, ${\mu }_{h}$ and Hall resistivity, ${\rho }_{h}$ measured at ambient temperature as shown in Table 3. All the thin film sample are the *n*-type conductivity with the negative average Hall effect coefficient values for each sample, implying electron as the major carriers in the film^49^. The thin film samples without the PTFE shows a significant increase in ${N}_{b}$ and reduction in ${\mu }_{h}$ and ${\rho }_{h}$ as the Cu nanoparticles concentration solution is raise (from TF-S1 to TF-S4) giving the surface area to volume ratio increase leading to increase surface activity^50^. Consequently, the density of free charge cariers transfer into the conduction band is enhanced. Thus, lower ${N}_{b}$ produces the highest ${\mu }_{h}$, and in this case, the variation of ${\rho }_{h}$ strongly depends on the ${\mu }_{h}$. The increase in the ${\mu }_{h}$ is attributed to the enrichment of crystalline quality and increase of the crystallite size. The larger crystallite size possesses lower grain boundaries density and hence increase in the crystallite size reduces grain boundary scattering and yield higher ${\mu }_{h}$. TF-S1 possesses higher ${\mu }_{h}$ (8.13 cm^2^·Vs^−1^) with ${d}_{m }\sim$ 20.3 nm. Conversely, the decrease in ${\mu }_{h}$ for TF-S4 (2.95 cm^2^·Vs^−1^) is ascribed to the degraded of the crystallite size with increase in multiply twinned particles. When the PTFE layer is added, the increase in number of micromolecule that in turn enhance the overcoat thickness of the thin film. Although the ${N}_{b}$ for TF-S3 + PTFE and TF-S4 + PTFE are decrease as compared to thin film without the PTFE, the ${\rho }_{h}$ is not significantly changing. These features are due to the homogeneous sealing up of defect and pores of the thin film. The PTFE layer improves the mobility and lowering the resistivity which reveal the significant contact between the layers.

## Conclusion

To summarise, Cu nanostructures was successfully synthesised via microwave-assisted technique with ultra-small ${d}_{m}$ Cu nanostructures. At this stage, synthesis parameters and types of reductants and surfactant with the correct amount of each are effective on the produced morphology to tune for the desired application. These Cu nanostructures take on a spherical shape consisting of innumerable Cu with the ${d}_{m}$ of 20.3 ± 1.4, 12.5 ± 0.6, 7.5 ± 1.4, 5 ± 2.2 nm for S1, S2, S3 and S4. The synthesised Cu nanostructures were then successfully deposited on PET substrate via the inexpensive, facile and rapid Mayer rod method. The Mayer rod method benefits from the ease of use, low cost, ease of processability and controllability also, a skilled person is not necessarily required for fabrication. The Cu nanostructures/PTFE-PET thin films show a low $\sigma$ compared to Cu nanostructures-PET thin films. The overcoat layer of PTFE offers the insulation of the coating, which decreases the amount of impurities penetrating the coating and the conducting surface is smooth. In this regard, PTFE seals up the defects and pores in the coating, which is attributed to the low $\sigma$ conductivity of Cu nanostructures. Upon analysing the different fabricated thin film, the Cu nanostructures with the PTFE layer does improves the mobility and lowering the resistivity as compared to the uncoated sample indicating successful protection thin film layer. Therefore, the work proposed further investigation in achieving a more efficient charge carrier transport to increase further the $\sigma$.

## Data Availability

The datasets used and/or analysed during the current study available from the corresponding author on reasonable request.
